# Pupil adjustments to illusory perceptions of the light intensity of object surfaces

**DOI:** 10.3389/fnhum.2025.1604114

**Published:** 2025-08-26

**Authors:** Bruno Laeng, Hüseyin Berke Canoluk, Shoaib Nabil

**Affiliations:** ^1^Department of Psychology, University of Oslo, Oslo, Norway; ^2^RITMO Centre for Interdisciplinary Studies in Rhythm, Time and Motion, University of Oslo, Oslo, Norway; ^3^Faculty of Medicine, İzmir Bakircay University, İzmir, Türkiye; ^4^School of Psychology, University of Sussex, Brighton, United Kingdom

**Keywords:** Cornsweet effect, illusion, brightness, lightness, luminance, surfaces, pupillometry

## Abstract

Using infrared eye tracking, we show that when gaze is maintained at the center of one of two equiluminant surfaces of a Cornsweet stimulus, designed by Lotto and Purves, that illusorily appear to be lighter or darker than the other, the eye pupils constrict or dilate, respectively. That is, pupil sizes mirror the subjective experience of differential brightness. Previous studies of pupil responses to illusions of light had focused on illusions of unveridical light sources (e.g., patterns resembling the sun), whereas in the present study, we show pupil adjustments to the illusory brightness of object surfaces within images of realistic scenes. In two control experiments, we also showed that when the edge gradients of the Cornsweet stimulus, which do differ in luminance, were either occluded or presented alone in a black field, there were no differences in pupil diameters. We also conclude that adjustments to the perception of surface reflectance are unlikely to represent anticipatory responses to probable risks of temporary visual impairment (i.e., dazzle to sunlight) and, instead, indicate that a gradual process of disambiguation of the visual scene is sufficient to elicit adjustments to the apparent light intensity of an object’s surface.

## Introduction

Our perception of the external world is intrinsically ambiguous; therefore, our brains must construct percepts that represent the probable causes of the sensory stimulations (e.g., Kersten et al., 2004; Brown and Friston, 2012). Light, or the visible range of electromagnetic radiations, can either stimulate our eyes directly from its sources (e.g., the sun, a fire, the pixels of a LED screen) or as a reflection from objects’ surfaces (e.g., the top of the table and the sea). However, our perceptions of the brightness (defined as the apparent intensity of light; see CIE, 1970; Blakeslee and McCourt, 2003) in the real world are not straightforwardly related to the physical parameters, as pointed out by several scholars (e.g., Purves and Lotto, 2003; Brown and Friston, 2012).

This uncomfortable fact, that we do not see reality as it is (a claim made by scores of philosophers and scientists; see Lotto, 2017), since we do not know directly the true causes of our perceptions, can be easily appreciated and effectively demonstrated by looking at optical illusions, i.e., particularly ambiguous or implausible scenes, where objective measurements can easily show that some of the scene’s different-looking objects (e.g., in either size, shape, color, or luminance) are in fact identical.

**Figure 1:**
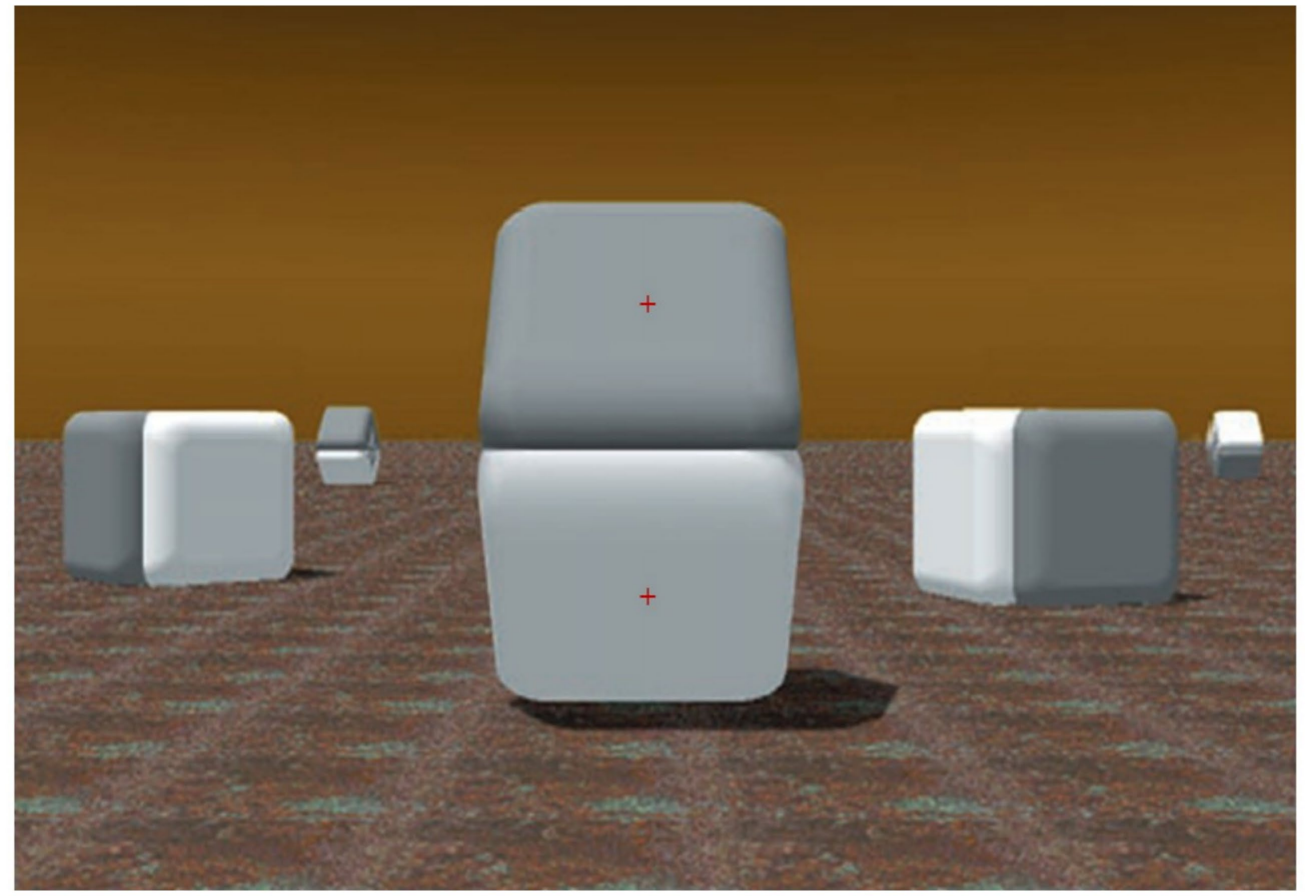
Illustration of the Craik–O’Brien–Cornsweet effect [image adapted with permission from Purves and Lotto (2003), courtesy of Beau Lotto and Dale Purves, https://www.americanscientist.org/article/why-we-see-what-we-do]. The centrally located shapes appear to have different brightness (i.e., darker on top). In each experimental trial, the red fixation cross appeared on only one of the surfaces.

Consider the Craik–O’Brien–Cornsweet effect (cf. Craik, 1966; Cornsweet, 1970; see also Adelson, 1993; Anderson et al., 2009; Brown and Friston, 2012), here exemplified in Figure 1 and used as a stimulus class in the present study. The scene in the image, designed by Beau Lotto and Dale Purves, represents what appear to be solid blocks lying onto the ground, or tilted and stacked on other blocks; crucially, the two surfaces appearing at the center of the scene are perceived, when looking at this image either on screen or printed on paper, having rather different brightness, and observers are unanimous in reporting that the top surface looks dark in relation to the whitish bottom surface (Purves et al., 1999, 2011).

**Figure 2:**
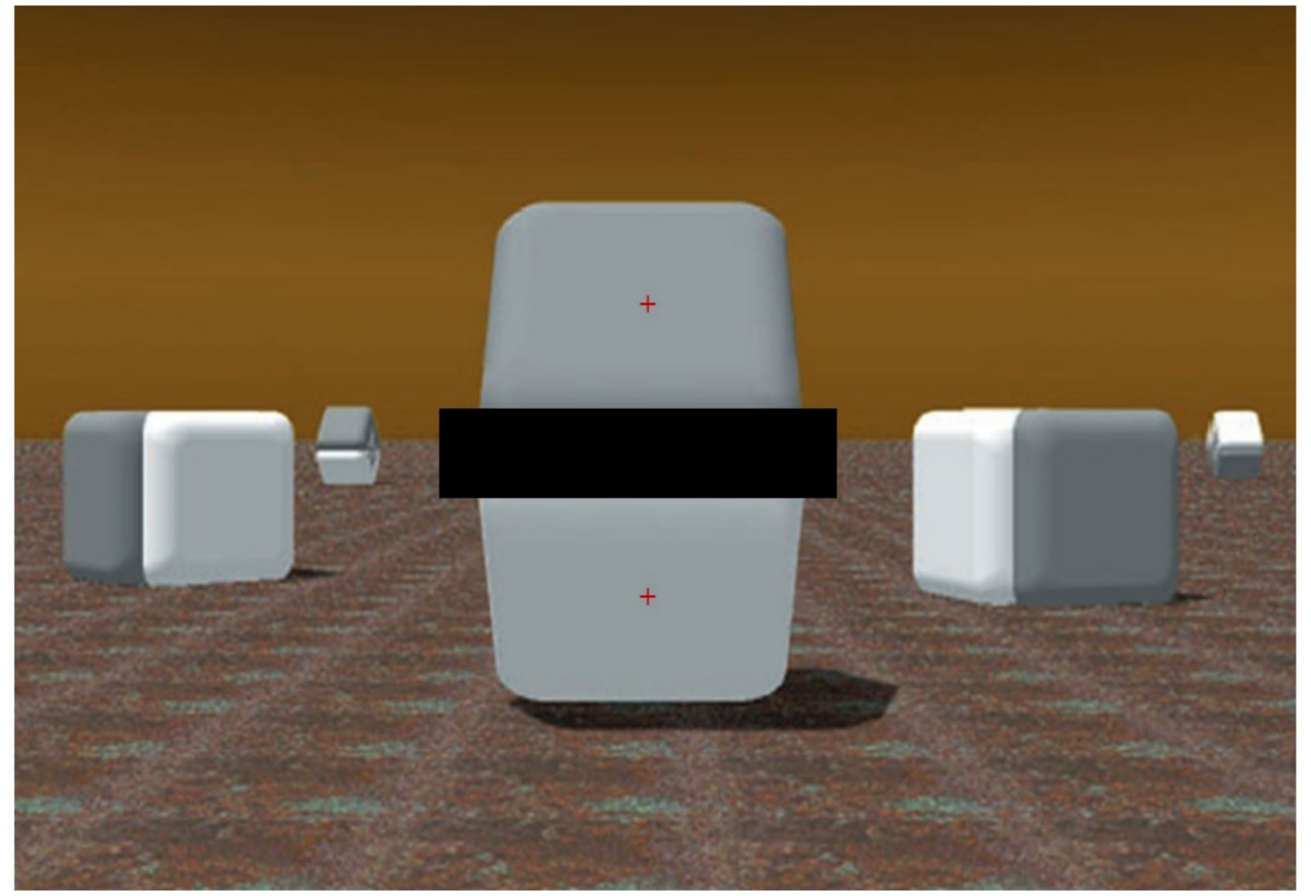
Same image of Figure 1 [adapted with permission from Purves and Lotto (2003)] with a horizontal dark bar masking the adjoining luminance gradients of the central two shapes. In contrast to the previous image, the two surfaces appear to have identical shades of gray (as they really are). In each experimental trial, the red fixation cross appeared on only one of the surfaces.

However, such a difference—since it is outside of the Cornsweet’s edge luminance gradients and appears to relate to the whole surfaces—is illusory. In fact, the pixels’ values (e.g., Luminance, Hue, and Saturation) over both the plane surfaces are identical, and, as Figure 2 shows, masking the luminance gradient regions, adjoining the surfaces of Figure 1, can nullify the effect and clearly reveal that the two whole surfaces do not differ in color at all.

The effect is caused by the presence in the image of a biphasic luminance ‘edge’ in the center of the scene (Brown and Friston, 2012). The images displayed here are compellingly strong examples of the Cornsweet effect. Indeed, mutually reinforcing, multiple cues are present over the whole image, including the periphery, that suggest the presence of different amounts of light falling on the target surfaces. Despite the two surfaces above and below the gradients of the Cornsweet’s edge objectively reflecting the same amount of light (or their pixels are equally bright on a screen), they subjectively look different. In this realistic-looking scene, light appears to be uniform and from the left, slightly from behind the target surfaces that appear to be stacked on the fronto-parallel plane, although slightly slanted in depth. The visible cast shadows reinforce the impression that light is from the top, as in a natural environment, and that due to the slanting surfaces, different light intensities may be reflected. Due to the cast shadow and a dark ground surface, the bottom surface is bordered below by a darker region than the empty background immediately above the top surface.

Several accounts have been proposed about the causes of such an effect in our visual system. The explanation offered in the seminal book by Cornsweet (1970) was based on considering physiological, spatial, and interaction in the visual system; that is, the filtering effects generated by lateral interactions among neurons at the visual input (e.g., in the retina). This explanation has been influential in accounting for illusory contrast effects (e.g., Sagi and Hochstein, 1985; Grossberg, 1987). More recent models (Dakin and Bex, 2003; Blakeslee and McCourt, 2003) have suggested that the mechanism responsible for the Craik–O’Brien–Cornsweet effect operates by amplification of the low spatial frequency structure of the image.

A series of previous studies have gathered evidence that the ocular response to the apparent intensity of light or *brightness* illusions is proportional to the perceived luminance of stimuli and not the actual light intensities on the retina (e.g., Laeng and Endestad, 2012), even when observers maintain the eyes’ foveae fixed onto a specific point over the image. Most studies have shown these effects to be robust, easily replicable, and with moderate to strong effect sizes, either in humans (e.g., Laeng et al., 2022, 2024; Zavagno et al., 2017), monkeys (Durand et al., 2024), or rodents (Vasilev et al., 2023; Saeedi et al., 2024).

Converging evidence has been obtained using stimuli that simply represented pictorially the sun (e.g., in photographs: Binda et al., 2013; Castellotti et al., 2020). Photos may be seen as more ‘ecological’ stimuli than the geometrical patterns designed by psychologists. However, the presence of luminance gradients (as in the abstract geometric pattern of the Asahi illusion by Kitaoka) reveals the core feature that triggers these pupil responses, and, in fact, the same luminance gradients are visible in the photos. Empirical evidence that pupil constrictions occur also to a cartoonish drawing of the sun (i.e., without the luminance gradients; cf., Naber and Nakayama, 2013) may suggest that semantic influences are sufficient to influence the pupils; however, these responses can just as well be accounted for by visual imagery being elicited by the pictorial representations (cf., Nanay, 2023). An imagery-based account has also been proposed for pupillary changes taking place when reading words referring to dark or bright things (e.g., Mathot et al., 2017). Indeed, other studies have found strong evidence that pupils constrict when participants simply imagine ‘looking at the sun’ (Laeng and Sulutvedt, 2014) or when they recall to mind objects just seen that differed in luminance (Kay et al., 2022).

Moreover, studies using the binocular rivalry method, where two differently luminant images alternate in awareness, have shown that the pupil diameters adjust to the luminance of the dominant perceptions and not to the combined luminance of the pair of images (e.g., Schütz et al., 2018; Naber et al., 2011; Acquafredda and Binda, 2024). Finally, several studies manipulating covert attention have shown that attending covertly (i.e., while fixating gaze somewhere else) on stimuli of different luminance modulates pupil size (as reviewed by Binda and Gamlin, 2017). The combined evidence of these studies leads to the conclusion that the oculomotor system’s adjustments to light are under the control of perception, attention, and imagery, instead of being a simple reflex mirroring the intensity scale of the luminance input (cf. Stark, 1959).

In relation to illusions of light, Dale Purves, Beau Lotto, and their colleagues (e.g., Purves et al., 2004) have singled out several cues as indicators of a high probability for a specific interpretation of a surface’s reflectance of light that are based on the assumption that perception is shaped by a lifetime experience with looking at objects’ surfaces under several transitions in the scene’s illumination. As Corney and Lotto (2007, p. 1790) specify: “By reflectance, we mean the proportion of incident light reflected by a surface; lightness is the perceived reflectance of a surface; brightness is the perceived intensity of light reaching the eye; and luminance is the actual intensity of the light that reaches the eye with respect to the sensitivity of the human visual system.” In other words, the brain’s spatial model of a visual scene, based on available depth cues, determines how lightness is assigned to each of the various surfaces that are present in the visual array (cf. Gilchrist, 1977). This process may occur rapidly and is probably accomplished by interactive processing between hierarchies of cortical and subcortical layers of the visual brain (cf., Anderson et al., 2009). Indeed, according to several accounts of perceptual decision under ambiguity (e.g., the ‘wholly empirical’ by Purves et al., 2011, or the ‘Bayes-optimal’ by Brown and Friston, 2012), the perception of relative brightness is predictably consistent with the combinations of three main parameters of light (i.e., illumination, reflectance, and transmittance) that have given rise to the same stimuli in past experience (of an individual or the species via natural selection).

Hence, ‘Cornsweet’ (in short) effects are far from being confined to just a few images designed to trick the eyes, but they are likely to be ubiquitous and common in daily visual experience. In general, we can think of optical effects and illusions as exposing the ecological constraints on our vision; that is, the prior experience with optical projections and light stimulation on the eye that observers typically obtain when moving through the world (Changizi et al., 2008; Changizi, 2009; Laeng et al., 2022, 2024). Despite the scene represented in Figure 1 being entirely static and depicted from a single point of view in two dimensions, the perceptual effect exposes the brain’s construction of the scene’s invariant properties, out of the original pattern of light intensities on the retina. Such a perceptual construction goes beyond the static, here-and-now information of the image and considers, based on prior experiences (Brown and Friston, 2012), possible future changes in projections (Freeman, 1994). Many perceptual changes occur as a consequence of our ‘active vision’, since we are highly mobile agents; hence, the brain’s visual system considers the likely direction of motion within the 3D space implied by the scene (Changizi et al., 2008).

That is, perception must constantly disambiguate what we see and assist the choice of the most appropriate behaviors (Purves et al., 2014). In the case of the Cornsweet effects, although the luminance of reflecting surfaces typically does not present a risk of incapacitating vision (although some exceptions exist: e.g., a prolonged exposure to the albedo of snow or water surfaces reflecting sunlight), we still expect that pupillary adjustments should occur based on the current perceptual representation as an optimal control of the bodily organs and behavior in relation to the observer’s ecology. Adjusting the pupil slightly (i.e., fractions of a millimeter, although corresponding to proportionally larger percentages of change in pupil area) yields visible changes in the luminance of a surface projected onto the fovea (Sulutvedt et al., 2021). Hence, showing that the pupil adjusts to the brightness of surfaces of these Cornsweet stimuli would represent not only a novel finding, but it would also strongly suggest that disambiguation of a visual scene is a sufficient reason for the control of the oculomotor system by illusory perceptions of light.

### The present study

We used the psychophysiological method of pupillometry to reveal how our brain constructs the properties of illusory light reflectance of objects’ surfaces, extending previous studies on the pupillary response to the illusory brightness of light sources. Specifically, we showed on a color screen two examples of Cornsweet effects, both digitally generated by Lotto and Purves and presented in several of their articles (e.g., Purves et al., 1999) or books (e.g., Purves and Lotto, 2003) or related commentaries (e.g., Morgan, 2003). The stimulus shown in Figure 1 had two target surfaces that are equiluminant but are seen as having different brightness. The masking shown in Figure 2 preserved the objecthood and volumetry of the original object in Figure 1.

In the present eye-tracking study, we presented repeatedly, for a few seconds, the scenes of Figure 1 (in Experiment 1) and Figure 2 (with the masked gradients and no Cornsweet effect, in Experiment 2), interspersed with some other filler images. In both experiments and every trial, gaze was initially maintained on a small red cross in the center of one of the equiluminant surfaces. We know from studies on the pupil light response that the adjustments of pupil diameter are principally governed by the central visual field (e.g., Kardon et al., 1991), as a consequence of pupil adjustments, being predominantly caused by retinal cones’ activity, in photopic or well-lit conditions.

Before each target image, a uniformly gray slide that was wholly equiluminant to the target surfaces in the target image allowed us to obtain a baseline measurement of the pupil. We monitored fixation with the eye tracker to ensure that participants kept their gaze on the cross at the center of each surface or of the baseline screen.

**Figure 3:**
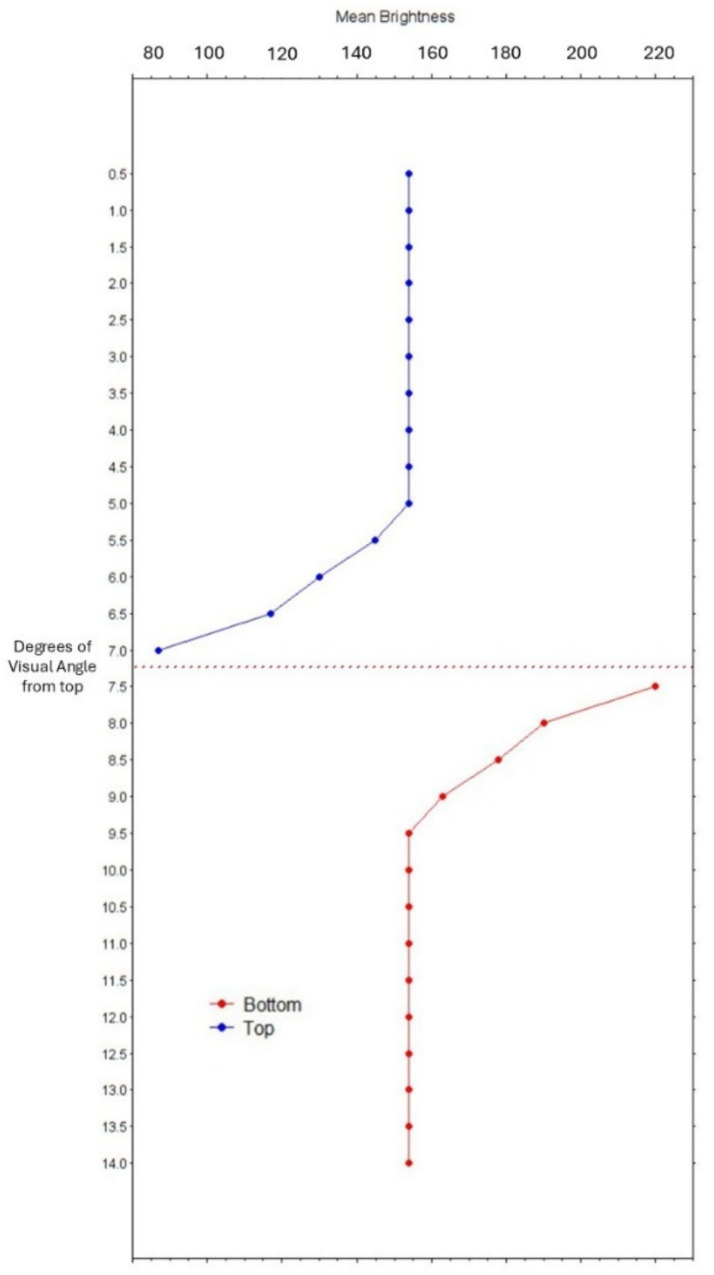
Graph of mean pixels’ brightness (x-axis) in steps of half a degree of visual angle from the top border of the two surface images (y-axis). The Cornsweet’s edge is located between 7° and 7.5° (dotted horizontal line).

We expected, based on the hypothesis of optimal control of oculomotor behavior, that the pupil diameters would be larger when viewing the gray-looking surfaces (on top in both stimuli of Figures 1, 3) than the white-looking surfaces (on the bottom in both stimuli) and, vice versa, would be relatively smaller when directly looking at the white-looking surfaces. In contrast, when looking at each of the surfaces (top or bottom) of Figure 2, we expected no significant differences in pupils’ sizes, since, in this case, all observers reported no experience of differential brightness or the Cornsweet effect, as in Figure 1.

## Methods

### Participants

Sixty-four participants (34 women; mean age = 28 years; *SD* = 8; 30 men; mean age = 35 years; *SD* = 12) were recruited at the University of Oslo, Norway, volunteering to participate anonymously in a perceptual study. All participants were tested in Experiment 1, and a subgroup of these (12 women; mean age = 27 years; *SD* = 7; 10 men; mean age = 31 years; *SD* = 14) participated in Experiment 2 as well. They were treated according to the Declaration of Helsinki, and the study was approved by the University’s IRB. All participants had normal vision or were corrected by contact lenses.

### Stimuli and apparatus

All stimuli consisted of the digitally generated images shown in Figures 1, 2, and the baseline image consisted of a gray field with a small red fixation cross (as shown in Figure 1). A color-calibrated Dell LCD monitor displayed the patterns at full screen with a resolution of 1,680 × 1,050 pixels. Each target surface of Figure 1 had a width of 6.95° in visual angles, with an average pixels’ luminance (in RGB scale) of 143.07. Figure 3 shows a graph of the change in pixels’ brightness from the top border of the image.

Pupil diameters were monitored at a sampling rate of 60 Hz with an infrared eye tracker (SMI R. E. D. 500). Experiment Center software (by SMI) controlled the presentation of trials, which was self-paced, allowing participants to proceed to the next trial by pressing a key when ready.

### Procedure

Participants were seated 65 cm from the screen, with the head stabilized in a chinrest. At the beginning of each block, they completed a standard 4-point calibration procedure. Each Cornsweet stimulus was presented full screen and for 4 s, whereas the baseline images were shown for 400 ms. Stimuli were presented in a fixed random order to every participant. There were six presentations of Figure 1 and six presentations of Figure 2; in half of these trials, gaze was forced on the cross in the middle of the top surface and on the bottom surface in the other half. All fixation crosses were in the same positions for baselines and target stimuli. Analyses from previous pupillometry studies with brightness illusions (e.g., Laeng and Endestad, 2012) confirm that six trials per stimulus type are sufficient for revealing pupil differences between the two conditions (i.e., dark vs. light).

## Results

We extracted each participant’s pupil diameters (in mm) in each trial using *BeGaze* software (SMI). Pupil data were based on pupil during fixations only, which excluded artifacts due to blinks or diameters during saccades. We used *BeGaze*’s function of delineating AOI (i.e., Areas of Interest) corresponding to the inner surfaces that were equiluminant but perceived with different brightness. This method excludes all fixations that occurred away from the fixations and outside the relevant target object’s surface. For the statistical analyses, we first averaged the pupil diameters in mm (grand mean = 4.56 mm, SD = 0.76; range = 2.8–7.1 mm) across all fixations that occurred within the relevant AOI during the presentation of a stimulus trial as well as during presentation of the baseline images (grand mean = 4.38 mm, SD = 0.76; range = 2.2–7.0 mm). No participants nor trials needed to be excluded, since the eye tracker captured all participants’ eyes for a percentage of time above 95%.

We then subtracted the average pupil diameter during baselines from the average pupil diameters when viewing the stimuli in the corresponding trials, obtaining a normalized measurement of pupillary change in mm (Laeng and Mathot, 2024). Such a normalizing procedure reduces the variance across participants by correcting for individual differences in baseline pupil diameter in an event-related manner, since we subtract pupil sizes measured right before seeing a stimulus from the pupil size when looking within the target surfaces’ AOI. In addition, this fixation-based procedure has the benefit of excluding all pupil measurements during saccades and artifacts due to eye blinks. The obtained average pupillary changes in each experiment were all analyzed with JASP software, using separate Bayesian sequential analyses to test the hypothesis (H1) that pupils adjust differently to the equiluminant surfaces, but also the null hypothesis (H0) of equal pupil sizes when viewing the control, masked stimulus in Figure 2.

### Experiment 1

**Figure 4:**
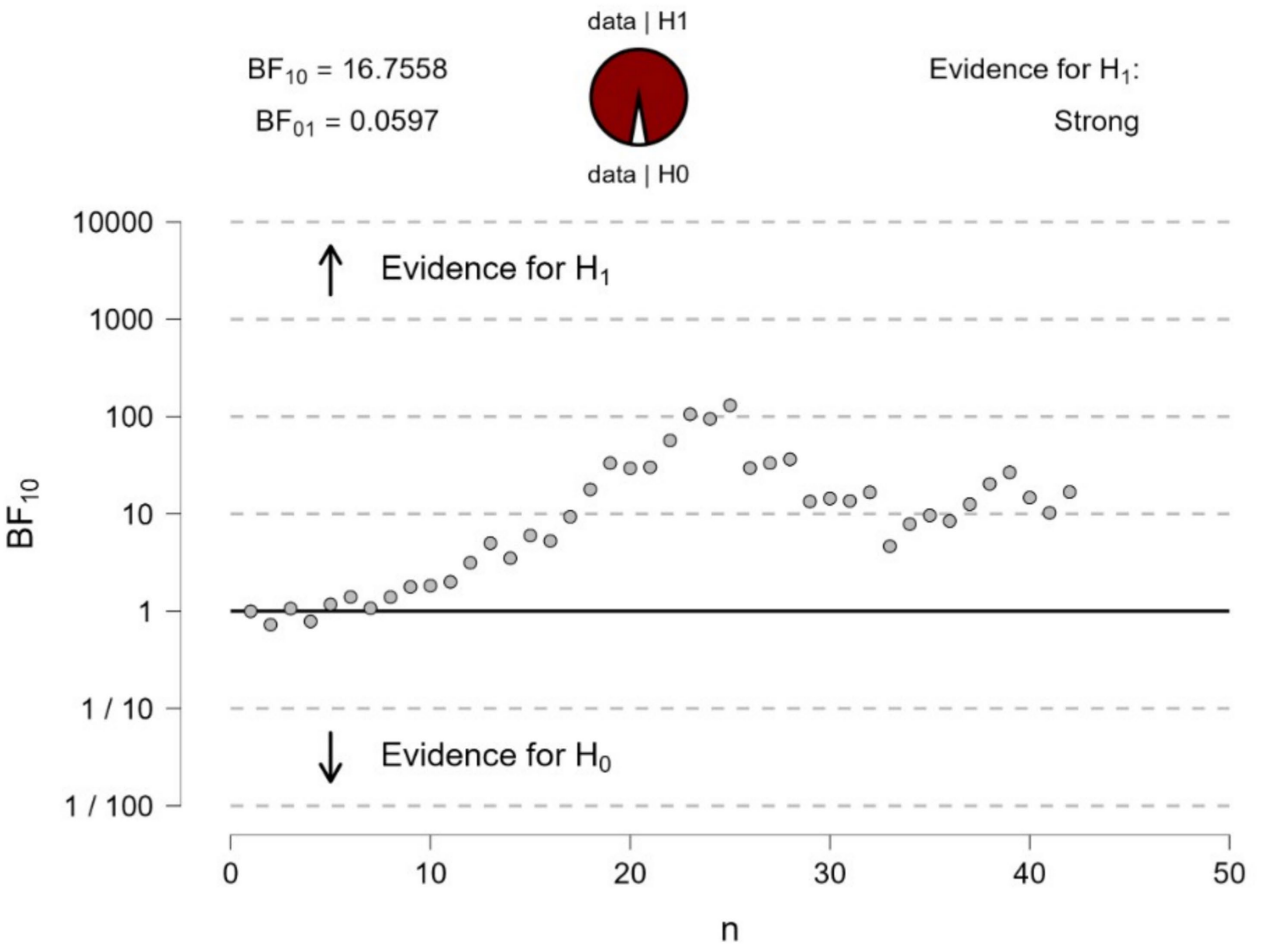
Bayesian sequential analysis of pupil changes to fixating at the center of the top or bottom surfaces (or illusory darker versus lighter) in Figure 1. The analysis was conducted using JASP software and displays both Bayes Factors (BF_10_ estimating the evidence in favor of H1—i.e., a difference in pupil diameters—and BF_01_ estimating the evidence in favor of H0—i.e., no difference in pupil diameters). Each circle in the graph represents the evidence contributed by a single participant according to the sequence of testing.

The Bayesian sequential analysis of the Cornsweet stimulus in Figure 1 yielded strong evidence (BF_10_ = 117.8) for a difference in pupillary responses when fixating on the top and bottom surfaces of Figure 1. The mean pupillary changes over time (in 200 ms epochs) over the ‘dark’ and ‘light’ surfaces are illustrated in Figure 4.

**Figure 5:**
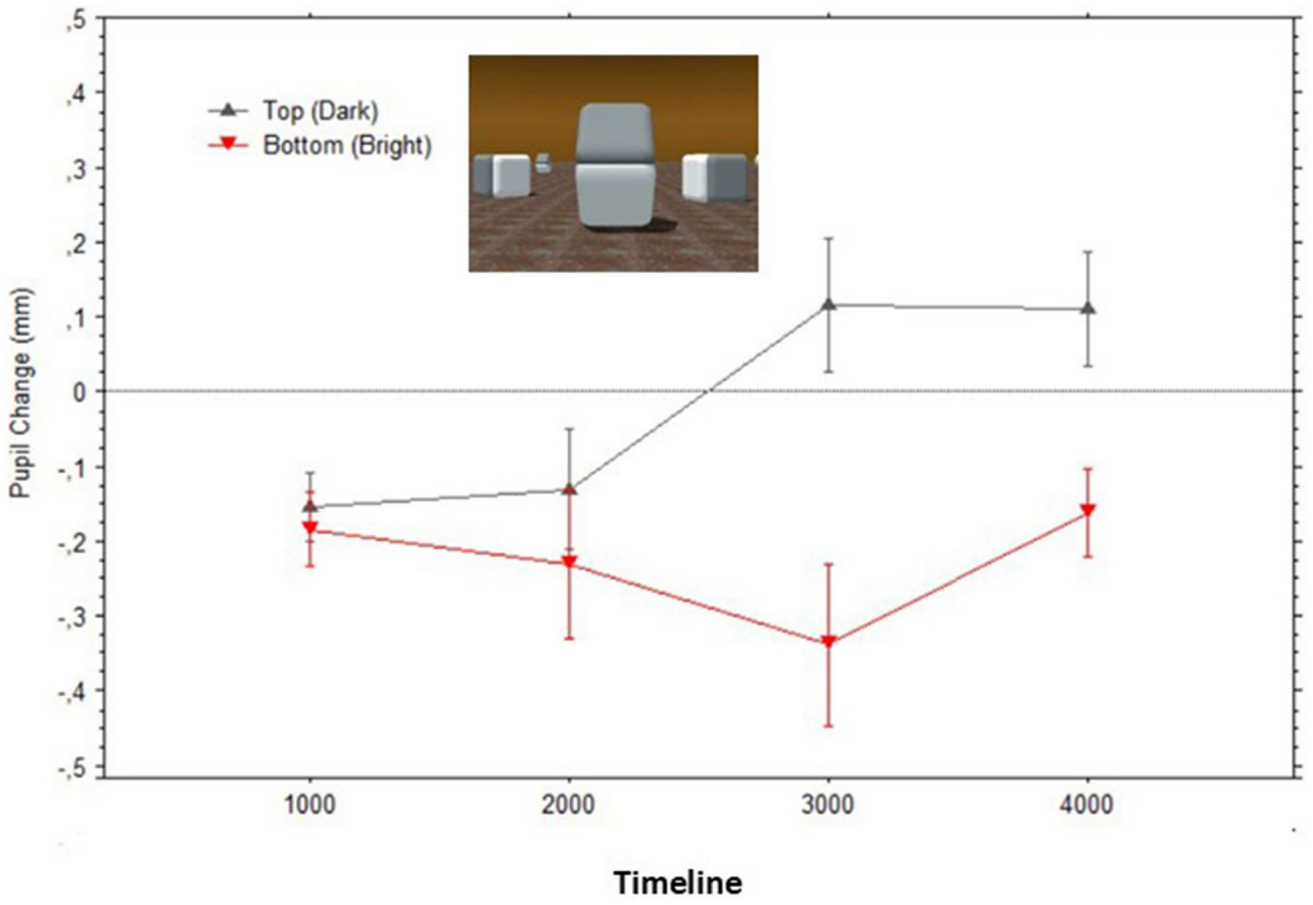
Mean pupil changes (in mm; bars show the Standard Errors) during the timeline of presentations of the stimulus in Figure 1 (averaged in epochs of 1,000 ms). Nota Bene: pupil diameters were obtained for each fixation within the AOIs corresponding to the target surfaces, and pupil diameters are plotted based on the fixations’ start time.

The pupils initially constricted to stimuli in relation to baselines, which is the typical pupil response when switching from a blank visual field (the baseline image) to a patterned scene (the Cornsweet); thus, signaling the optical focusing response of the eye (Barbur, 2004). Interestingly, as visible in Figure 5, first the pupil diameters to the surfaces were very similar to each other and to the response to the equiluminant baseline image, and only after looking at the image for a couple of seconds, the pupils dilated above the baseline level for the illusory ‘dark’ surface or constricted below the baseline for the illusory ‘light’ surface.

### Experiment 2

**Figure 6:**
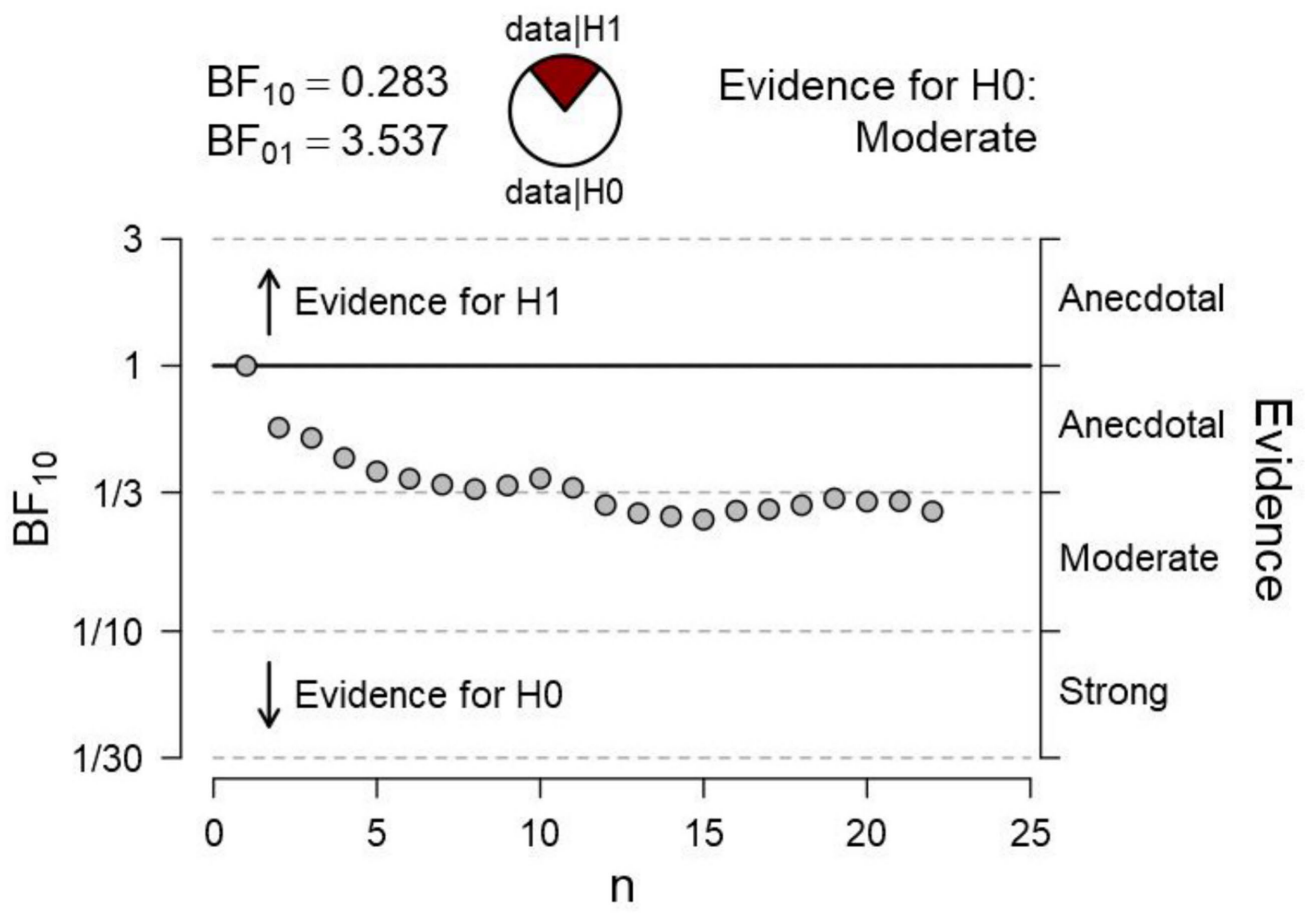
Bayesian sequential analysis of pupil changes to the surfaces with masking of adjacent gradients in Figure 2. The analysis was conducted using JASP software and displays both Bayes Factors (BF_10_ estimating the evidence in favor of H1—i.e., a difference in pupil diameters—and BF_01_ estimating the evidence in favor of H0—i.e., no difference in pupil diameters). Each circle in the graph represents the evidence contributed by a single participant according to the sequence of testing.

In a second experiment, we tested the hypothesis that, when the adjoining gradients of the surfaces are occluded (i.e., by masking the gradients, as in Figure 2), there would be no difference in pupil size, also consistent with the phenomenological report of an identity in the surfaces’ brightness. Bayesian statistics is particularly appropriate (Dienes, 2014) to gather conclusive evidence in favor of the null hypothesis. A t-test sequential analysis (with JASP software^1^) of the paired comparison of pupillary changes to each of the surfaces provided moderate evidence in favor of the null hypothesis. As recommended in the Bayesian statistics literature on t-test sequential analyses (e.g., Schönbrodt et al., 2017; Mani et al., 2021), we terminated data collection after the Bayesian Factor reached an asymptotic level. As shown in Figure 6, an asymptotic BF value and moderate evidence in favor of H0 were reached (BF_01_ = 3.537) after testing about half of the sample size used in Experiment 1. This evidence led us to conclude in favor of no difference between diameters when the gradients were occluded from sight.

**Figure 7:**
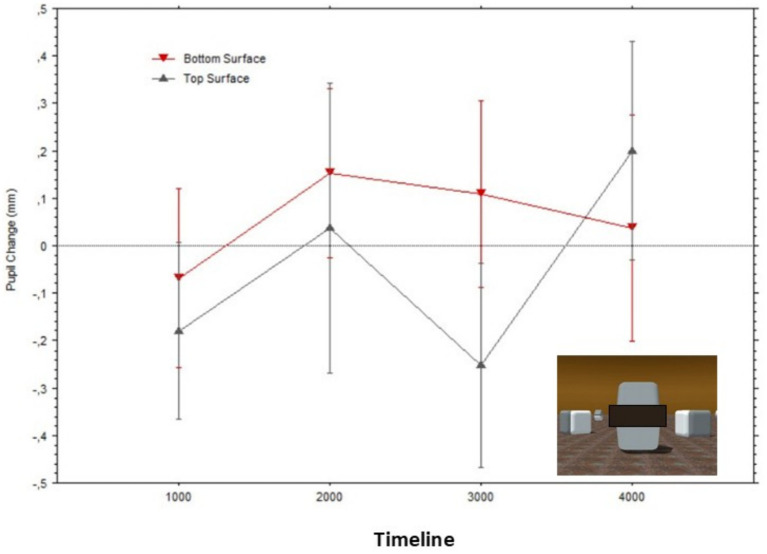
Mean pupil changes (in mm; bars show the Standard Errors) during the timeline of stimulus presentations (averaged in epochs of 1,000 ms) for the ‘masked’ surfaces of the stimulus in Figure 2 (also shown in inlet). Pupil diameters were obtained for each fixation within the AOIs corresponding to the target surfaces, and pupil diameters are plotted based on the fixations’ start time.

Figure 7 illustrates how the average pupil diameters, when fixating gaze on each surface, greatly overlapped while observers looked at what originally were the bright versus dark surfaces of the Cornsweet stimulus; Top surface: Mean = −0.22 (SD = 0.27), Bottom surface: Mean = −0.18 (SD = 0.32).

### Experiment 3

In a final experiment, we considered the possibility that the region of the edges’ gradients, which do differ in luminance and are essential for the illusory brightness of the whole surface to be generated (as shown in Experiment 2), could be sufficient to yield the observed pupillary adjustments (as shown in Experiment 1), despite the fixation points being several degrees away from these edges. In other words, though the illusory brightness of each surface appears over its whole extent, including the region at fixation, it is still possible that the observed smaller pupil size was due to the difference in luminance between the bright edge to the point of fixation on the lower surface and the darker edge of the upper surface.

Previous psychophysical studies (for a review, see Watson and Yellott, 2012) have shown that pupillary constrictions are evoked by light stimulation across a large portion of the visual field. For example, Stanley and Davies (1995) showed that pupil size was dependent on the product of luminance and adapting field size, showing that the diameter could constrict by several millimeters more when changing the field diameter from 0.4° to 25.4°. Specifically, Stanley and Davies (1995) started with central vision or 0.4° (i.e., about the size of the foveola) and, based on their graph (p. 602, Figure 1), pupils constricted (e.g., with about 100 cd/m^2^ luminance) by 15% more with a 1.6° field (about the size of the fovea) and 67% more with 7.4° field (a bit larger than the perifovea). Given the increases in constrictions with the increasing extent toward the peripheral regions of the visual field, we surmise that this response reflects an adjustment to illuminance (i.e., the total “amount” of visible light) over the visual field. Moreover, such a response could be aided by the additional responses of melanopsin-expressing ganglion cells (also ipRGC) in the primate retina, which are activated by both the rods and cones causing pupil constrictions (Spitschan et al., 2014); however, note that the receptive field of these cells are very large (Dacey et al., 2005) and integrate large portions of the visual field. Although the ipRGC is slower than the one mediated by photoreceptors such as cones (Tsujimura and Tokuda, 2011), it is likely to occur within the presentation times that we used in the present experiments. Hence, it is a possibility that the pupillary changes we observed may have been affected differentially by the luminance gradients, depending on which luminance gradient (bright/dark) was closer to the fixation point.

However, we need to consider the possibility that in photopic, well-lit conditions in which the retinal rods are inactive, pupil adjustments to light energy might reflect predominantly the stimulation of the retinal cones (e.g., Barrionuevo et al., 2014). We also consider the fact that only approximately 15% of the retinal cones are located outside of the fovea and parafovea (Wandell, 1995). Indeed, Mizukawa (2009, p. 54, Figure 3) stimulated the eye with high contrast white patches (100 cd/m^2^) of approximately 5°, starting from central vision and displacing them until 15° from central vision, and found that the pupil constriction with stimuli immediately surrounding the central region had reduced to a 16% of its amplitude with central stimulation. Thus, we surmise that—in the present experiments—when maintaining fixation on the cross, the Cornsweet’s edge gradients would fall outside the retinal area that contains the most responsive region of the visual field. We also need to consider that the ganglion cells’ receptive fields often reflect the activity of single cones in the fovea. However, outside the fovea, several adjacent cones’ activity are summed together, causing ganglion cells’ receptive fields to progressively increase in size in relation to increasing peripheral positions (Ahmad et al., 2003). The large receptive fields of peripherally located ganglion cells, as well as the large receptive fields of the non-image forming ipRGC, would be likely to average together both luminance edges of the Lotto and Purves’ Cornsweet stimulus, while keeping fixations on the positions used in the present experiments, possibly reducing out the individual luminance effects of each gradient edge from the points of fixation.

**Figure 8:**
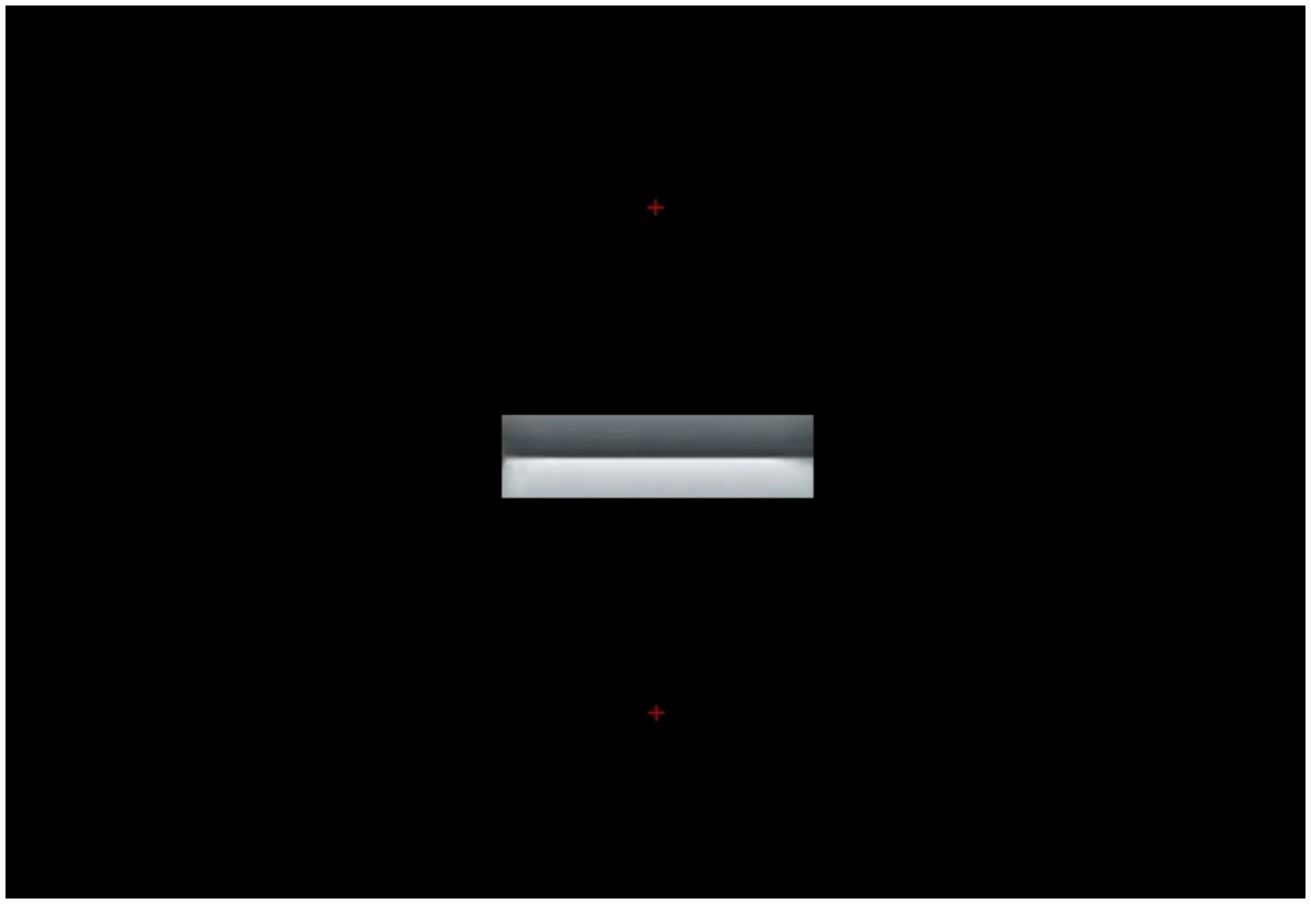
Edge gradients of the Cornsweet’s image in Figure 1 are shown alone, the rest of the scene being replaced with a black background. In each experimental trial, one small, red, fixation cross appeared either on top of or below the edges (both shown here for illustration).

Hence, it is really an empirical question whether, in the specific conditions of our experiment, the two gradient segments (each extending about 2° vertically and 15° horizontally) would be sufficient to drive the pupil differences when fixating either above or below the edge gradients as shown in Figure 1. To put to test this alternative hypothesis, we presented in a third (control) experiment, the image shown in Figure 8, presenting the fixation points in the same screen positions of the previous two experiments, while blackening out the whole scene except for the two edge gradients of the original Cornsweet stimulus.

### Participants

Thirty-five participants (18 women; mean age = 27 years; *SD* = 9; 17 men; mean age = 29 years; *SD* = 11) were recruited at the University of Oslo, volunteering to participate anonymously in a perceptual study. All participants had normal vision or were corrected by contact lenses.

### Stimuli, apparatus, and procedure

All stimuli consisted of the images shown in Figure 8, with half of the trials showing either the upper fixation cross or the lower one. As before, the baseline image consisted of a gray field with a small red fixation cross in the same position and the average luminance of the following stimulus. We used the same apparatus as in Experiments 1 and 2 with the same registration parameters. Pupil diameters were monitored at a sampling rate of 60 Hz with an infrared eye tracker (SMI R.E.D. 500). Experiment Center software (by SMI) controlled the presentation of trials, which was self-paced, allowing participants to proceed to the next trial by pressing a key when ready. We also used the same procedure as the previous two experiments, where the target stimulus was presented full screen for 4 s, preceded by the baseline image for 400 ms. Again, there were six presentations for each fixation cross position.

## Results

As done earlier, we extracted each participant’s pupil diameters (in mm) in each trial using *BeGaze* software (SMI), based on pupil diameters (in mm) during fixations only, excluding artifacts due to blinks or diameters during saccades. We delineated circular AOIs corresponding to each fixation point with a diameter corresponding to about 1° of radius to exclude fixations outside of the fixation regions. We first averaged the pupil diameters in mm as well as during presentation of the baseline images. No participants nor trials needed to be excluded, since the eye tracker captured all participants’ eyes for a percentage of time above 95%.

**Figure 9:**
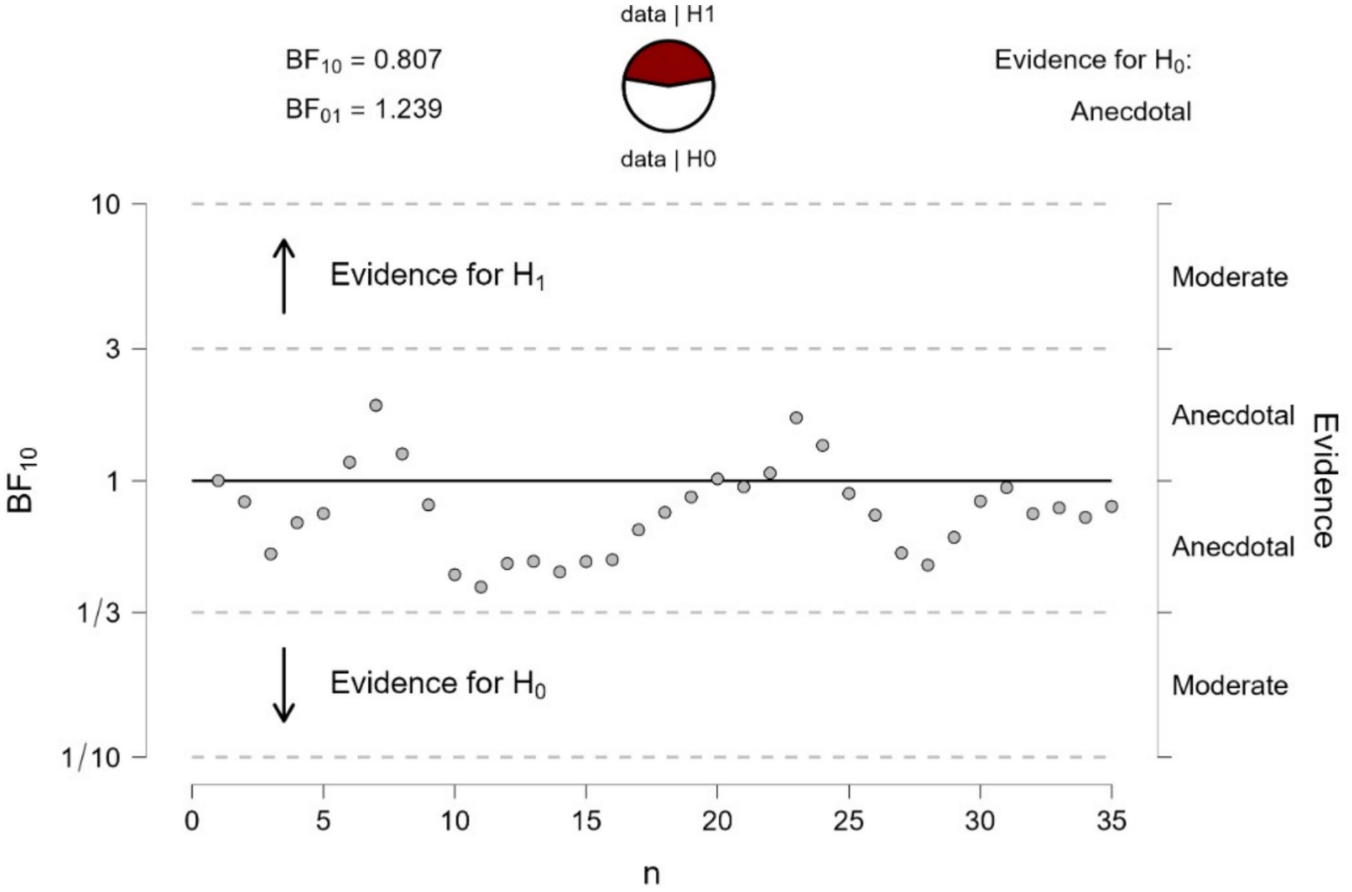
Bayesian sequential analysis of pupil changes to the gradients of Figure 8. The analysis was conducted using JASP software and displays both Bayes Factors (BF_10_ estimating the evidence in favor of H1 and BF_01_ estimating the evidence in favor of H0; in the present case, there was no conclusive evidence for either H1 or H0). Each circle in the graph represents the evidence contributed by a single participant according to the sequence of testing.

We run a Bayesian sequential analysis on these data (see Figure 9), which indicated anecdotal evidence in favor of the null hypothesis (BF_10_ = 0.81). The BF values showed a nearly asymptotic profile remaining below BF = 1 for most of the sequential testing. Hence, the results obtained when participants looked at the control stimulus in Figure 8 were very different from those when looking at Figure 1, which had shown extreme evidence in favor of a different pupil response.

**Figure 10:**
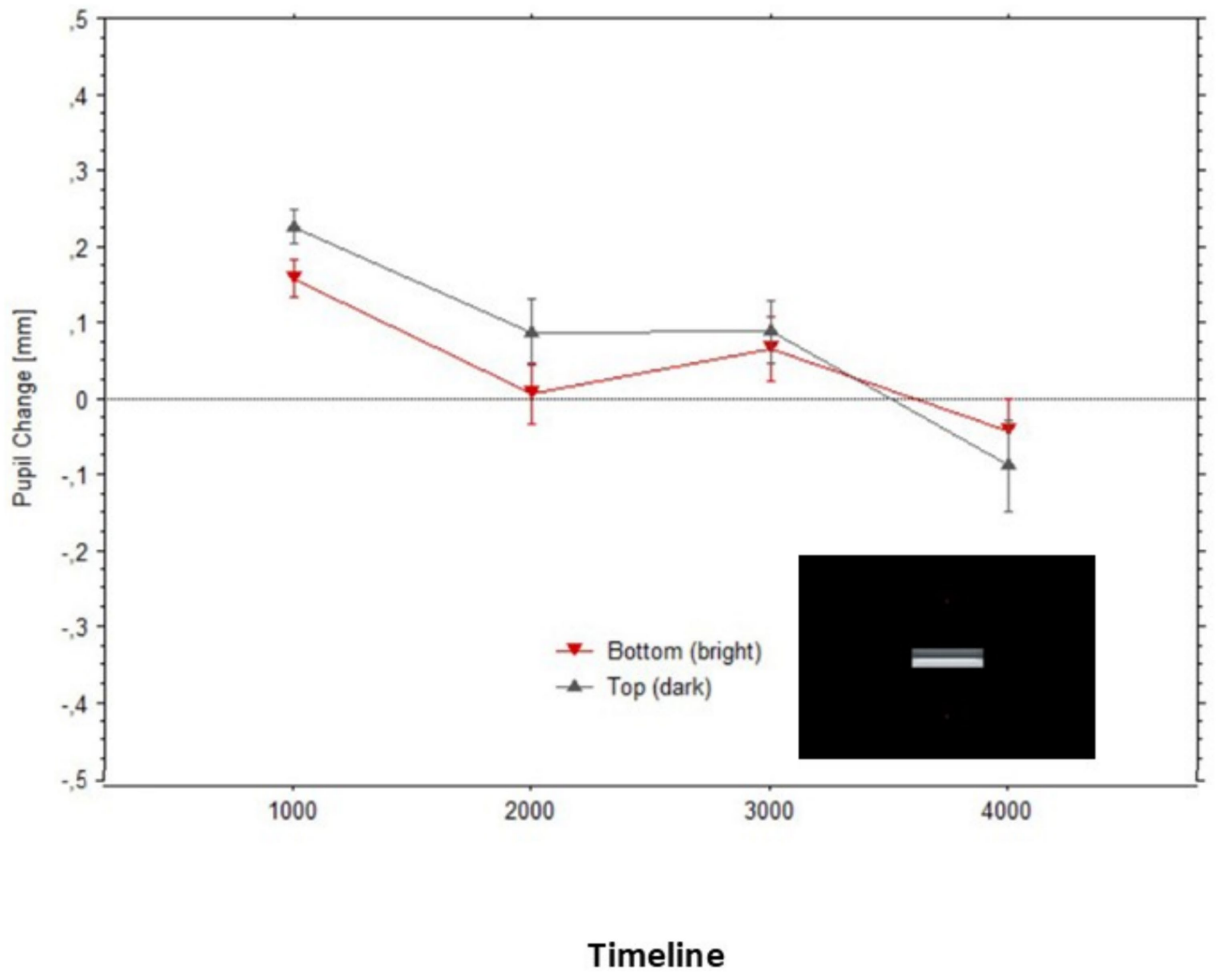
Mean pupil changes (in mm; bars show the Standard Errors) during the timeline of presentations of the stimulus in Figure 8 (averaged in epochs of 1,000 ms). Pupil diameters are plotted based on the fixations’ start time.

The mean pupillary changes over time (in 1,000 ms epochs) are illustrated in Figure 10, split by fixations either above or below the two edges. The pupils appear to initially dilate in both fixation conditions, in relation to baselines (Figure 10), probably due to the overall darkness of the stimuli. Then, the pupil diameters progressively constricted in both conditions until, after approximately 2 s, they became very similar to each other. Although Figure 10 may give the impression that during the first epoch (1,000 ms), diameters were smaller for the bright edge compared to the dark edge, a paired t-test confirmed that these diameters did not differ significantly, t(13) = 0.11, *p* = 0.87.

Overall, the changes in pupil diameters in Experiment 3 were clearly reduced compared to what we observed in Experiment 1. Whereas the maximum average difference between pupil dilations (to the illusory darkness) and the relatively constricted pupils (to the illusory brightness) reached 0.41 mm in Experiment 1, it did not exceed 0.1 mm in Experiment 3.

## Discussion

The brightness of the two central surfaces of the Lotto and Purves’ Cornsweet stimulus used in the present experiment looks to observers as uniformly being the same, despite only the adjacent, central, horizontal edges differing in luminance. What we showed here is that, when centering gaze on a region of each surface that emitted the same amount of light, the pupils’ diameters adjusted to the perceived brightness of the surfaces and not to the objective pixels’ luminance intensities. Specifically, pupil diameters were on average smaller when fixating on a surface appearing to be brighter than an equiluminant surface but darker in appearance. These pupillary changes to the ‘dark or light’ surfaces’ brightness evolved within the first 2 s of perceiving the surface and the scene. Importantly, two control experiments conjointly showed that the Cornsweet effect on the pupil disappeared when (a) masking only the adjoining gradients in the Cornsweet stimulus (Experiment 2), despite gaze was directed to the same fixation points on the surfaces of both the unmasked and masked stimuli and (b) when showing only the adjoining gradients of the original Cornsweet stimulus (Experiment 2), centered in the same position but within a black field. The pupil adjustments in Experiment 1, where the surfaces were perceived as different in brightness, could be 4 times greater than in Experiment 3, where only the edges were shown within a dark field. Clearly, based on these findings, the pupils adjusted to the perceived brightnesses of each surface, and they did not simply reflect the luminance difference between the gradient edges.

These findings were expected by an account where pupillary adjustments are controlled by the perceptual (subjective) experience of brightness and not exclusively by the physical (objective) light energy on the retina. The two control experiments lead to the conclusions that (a) the gradient edges had a causal effect in the subjective brightness perceived over the whole surface belonging to each gradient, but (b) that the luminance difference between the two gradients was not sufficient in accounting for the pupil adjustments measured during the illusory perception. A comparison of Figures 5, 10 reveals that the average magnitude of change of the pupil diameters could be four times larger when fixating on the illusory surfaces than when only the gradient edges were visible at the same distances from fixation.

One possible account of the Cornsweet effects and the co-occurrent pupil adjustments, based on current accounts of predictive perception (Purves et al., 2011; Brown and Friston, 2012), is that these reveal the visual system’s prior experiences with (curved) edge gradients of visual stimuli seen under differing illumination conditions in real-world scenes. Such a perception, albeit unveridical and illusory when compared to actual physical information registered by the sense organs, provides a better spatial model that optimizes possible interactions, or affordances, with these edges.

Previous studies on illusions of brightness used mainly very abstract stimuli and illusions consisted of seeing (illusorily) a source of light, or its absence, but not light emitted from an object’s surface, as in the present study. For example, with the Asahi image in Laeng and Endestad (2012), observers typically see an illusory bright region (where gradients converge) that does not appear as being reflected by any surface but occurring in empty space. Similar to the expanding dark holes illusion (Laeng et al., 2022, 2024), the illusory darkness is not part of an object but of an empty hole. Except for the Kanizsa illusion (also used in the 2012 study of Laeng and Endestad), where the illusion of a surface that is brighter than the background happens on what is itself an illusory surface (induced by the convergence of the ‘pacmen’), it appears that all the previous studies have not explicitly dealt with the brightness of depicted objects’ surfaces.

What is compelling about the present image by Lotto and Purves, used in the present study, is that it represents a scene in a manner very close to seeing a real-world scene, with the variety and complexity of perceptual cues, and three-dimensional object surfaces. Interestingly, Purves et al. (2004) pointed out that, both in ordinary usage and in visual psychophysics, we can distinguish between *brightness* and *lightness*; in their account, the former refers to the apparent intensity of light that can be attributed to a primary source of light (e.g., the sun, a lamp), whereas the latter refers to the apparent intensity that is the consequence of surface reflectance. This distinction is relevant here, since there is a gap in knowledge with regard to illusory surface reflectance, as defined above, and the terms describe different properties of light in the real world, which are linked to different expectations and behavioral consequences.

Incidentally, the present study revealed a gradual change (taking approximately 2 s from image onset) in the pupil adjustments to the illusory percept, relatively to those observed in previous studies (e.g., Zavagno et al., 2017; Suzuki et al., 2019). We can only speculate that the delay in processing measured in this study may be due to the scene’s complexity (e.g., presence of multiple surfaces, shadows, depth, and direction of lighting), which influences the perceptual construction of the whole scene, especially in conditions of forced fixation. Indeed, when gaze fixation is on the crosses, the whole scene will be seen in low resolution, and only the two identically bright regions would be seen in high resolution. Thus, the delay may be due in part to the initial adjustment of focus, but over time to the gradual perceptual construction of the whole scene.

In addition, an important consequence of comparing the present results with those of previous experiments with illusory brightness (e.g., Zavagno et al., 2017; Laeng and Endestad, 2012; Suzuki et al., 2019) or, its opposite, darkness (e.g., Laeng et al., 2022, 2024) is that a previous account of the illusory perception of brightness and associated pupil adjustments, as anticipatory responses to risks and uncertainties about sudden increases or decreases in brightness, cannot be invoked for the present results. Although, for some of these illusions, the pupillary adjustments to illusory brightness or darkness might represent adaptations, likely to reduce the risk of being unable to see in the next moment (e.g., risks of collisions, failed detection of a danger), it seems unlikely that similar mechanisms underlie the pupillary adjustments to the illusory *brightness* of surfaces since their reflectance rarely puts us at risk (although there is a ‘glaring’ exception with the ‘snow-blindness’ due to excessive exposure to snow’s albedo). Therefore, the present pupillary adjustments not only extend the range of illusory features that influence the eye pupils, but strongly suggest that the process of disambiguating surface light properties in the visual scene is a sufficient reason for eliciting pupil adjustments.

In general, the pupillometry research of recent years has pointed to a continuum between perception and illusions (Laeng and Endestad, 2012; Binda et al., 2013; Laeng et al., 2022, 2024) and mental images (e.g., Laeng and Sulutvedt, 2014; Pearson et al., 2015). For example, generating a mental image of common objects, at a certain distance and size (e.g., a pencil at 4 meters), triggers an oculomotor near-response (i.e., changes in both the vergence of the eyes and diameters of the pupils; Sulutvedt et al., 2018), despite what is imagined is not what is sensed by the eyes at the same time (i.e., in this experiment: a blank computer screen). Indeed, percepts and mental images derive from the activity of the same brain regions (Kosslyn, 1994) and obey the same powerful constraints (Moulton and Kosslyn, 2009) for resolving perceptual ambiguities and optimizing behavior (Changizi et al., 2008).

## Data Availability

The raw data supporting the conclusions of this article will be made available by the authors, without undue reservation.

## Footnotes

^1^ https://jasp-stats.org/
